# Role of adipocytokines in endometrial cancer progression

**DOI:** 10.3389/fphar.2022.1090227

**Published:** 2022-12-12

**Authors:** Ran Li, Fang Dong, Ling Zhang, Xiuqin Ni, Guozhi Lin

**Affiliations:** ^1^ School of Health Sciences, Jiangsu Food and Pharmaceutical Science College, Huaian, China; ^2^ Department of Obstetrics and Gynecology, Second Affiliated Hospital to Shandong First Medical University, Taian, China

**Keywords:** endometrial cancer, signalling pathway, adipokines, inflammatory cytokines, angiogenic factors

## Abstract

Endometrial cancer is considered a significant barrier to increasing life expectancy and remains one of the most common malignant cancers among women in many countries worldwide. The increasing mortality rates are potentially proportional to the increasing obesity incidence. Adipose tissue secretes numerous adipocytokines, which may play important roles in endometrial cancer progression. In this scenario, we describe the role of adipocytokines in cell proliferation, cell invasion, cell adhesion, inflammation, angiogenesis, and anti-apoptotic action. A better understanding of the mechanisms of these adipocytokines may open up new therapeutic avenues for women with endometrial cancer. In the future, larger prospective studies focusing on adipocytokines and specific inhibitors should be directed at preventing the rapidly increasing prevalence of gynecological malignancies.

## 1 Introduction

Endometrial cancer is considered a significant barrier to increasing life expectancy with significantly increased incidence (Morice et al., 2016) and remains one of the most common malignant cancers among women in many countries worldwide, particularly in more developed countries (Oaknin et al., 2022). Worldwide, endometrial cancer, which is classified into two histological subtypes (type I and type II), ranks sixth in incidence among all female cancers (Morice et al., 2016; Sung et al., 2021). Data from the International Agency for Research on Cancer indicate that 417,367 new corpus uteri cancer cases and approximately 97,370 deaths occurred in 2020. According to the results of previous study, the highest incidence rate was noted in North America (21.1 per 100,000) and was approximately 10-fold greater than the lowest rate, which was observed in Middle Africa (2.3 per 100,000) (Sung et al., 2021).

However, the variation in mortality rates in different regions was not as obvious. The lowest mortality rate was observed in Northern Africa (0.7 per 100,000), and the highest was noted in Eastern Europe (3.7 per 100,000) (Sung et al., 2021). In China, the endometrial cancer incidence rate is approximately 7.74/100 000, and the mortality rate is approximately 1.60/100 000 (Sun et al., 2022). The increasing mortality rates are mainly associated with the increasing incidence of obesity, a leading cause of endometrial cancer (Ding et al., 2020; Larsson.et al., 2022; Moukarzel et al., 2022). In adjusted mixed linear models, weight loss is strongly related to the levels of cancer-associated biologically active substances, including reduced interleukin-6 (IL-6) levels and increased adiponectin levels (Linkov et al., 2012).

As a major site for the secretion of protein signals, adipose tissues mainly comprise adipocytes. In addition, as a major endocrine gland, dysfunctional adipose tissue is involved in obesity-related tumorigenesis, which is correlated with its high degree of plasticity (Sakers et al., 2022) and the permissive microenvironment generated by aberrant inflammatory cytokines, adipokines, angiogenic factors, and aromatase (Hefetz-Sela and Scherer 2013). White adipose tissue (WAT), the most abundant adipose form, secretes numerous adipokines and cytokines to regulate whole-body metabolism. Moreover, WAT inflammation, which increases the expression of proinflammatory and proneoplastic genes, is associated with endometrial cancer (Moukarzel et al., 2022). Additionally, it has become helpful to evaluate biomarkers in relation to cancer risk (Linkov et al., 2018).

## 2 Article types

Review.

## 3 Manuscript formatting

### 3.1 Role of adipokines in endometrial cancer progression

Adipokines, a diverse group of biologically active substances, are characterized by adipose tissue secretion (Trayhurn and Wood 2004). The levels of various adipokines, such as leptin (Madeddu et al., 2022), visfatin (Wang et al., 2019), galectin (Boutas et al., 2021), resistin (Ozgor et al., 2019), adiponectin (Ellis et al., 2020), and vaspin (Erdogan et al., 2013), are increased or decreased in endometrial cancer and significantly correlated with cancer progression (Ray et al., 2022).

#### 3.1.1 Leptin

Leptin is a 16 kDa cytokine-like hormone encoded by the obesity gene on chromosome 7q31.3, which was first discovered in 1994 (Zhang et al., 1994). The mature leptin protein consists of 146 amino acids and is mainly secreted from white adipose tissue (Zhang et al., 1994). Women with genotype AG of SNP -2548 G/A of leptin are less likely to be at risk for endometrial cancer given that the heterozygote AG is less frequently observed in endometrial cancer patients (Bienkiewicz et al., 2017). Leptin acts on the hypothalamic regions by binding to leptin receptors (Ob-R), which exist in six isoforms with different lengths and C-terminal sequences (Baumann et al., 1996). The AG polymorphic variant of SNP LEP-R c.668A>G (p. Gln223Arg, rs1137101) in the leptin receptor is less frequently observed and considered a protective factor in women with endometrial cancer (Bienkiewicz et al., 2021). By analyzing data from tissue samples and whole blood, overexpression of leptin and its receptors was implicated in endometrial cancer both at the mRNA and protein levels (Boron et al., 2021). In endometrial cancer tissues, Ob-Ra is considered the most common form influencing biological outcomes, not Ob-Rb, which has the same extracellular domain (Yuan et al., 2004). Expression of the long leptin receptor isoform is approximately 5-fold higher in neoplastic tissue compared with normal tissue (Mantzos et al., 2011).

Leptin is involved in endometrial cancer by controlling energy homeostasis and increasing glycolytic capacity. Exposure to leptin could alter endometrial cancer cell morphology. The higher the leptin concentration, the greater the surface roughness (Dabrus et al., 2020). A positive correlation was noted between endometrial cancer and elevated serum leptin levels (Petridou et al., 2002; Tessitore et al., 2004). The incidence rate increased with increasing body mass index (BMI) in endometrial cancer patients (Cymbaluk et al., 2008). The cancer risk of postmenopausal women with the highest tertile of circulating leptin levels was almost three times that noted for women with the lowest tertile (Dallal et al., 2013). In addition, overexpression of leptin and its receptors was observed (Boron et al., 2021). These observations indicated that leptin and its receptors may be potential targets for intervention in the pathophysiology. Furthermore, useful cancer treatment strategies could be designed based on these findings (Boron et al., 2021).

To understand the potential molecular mechanisms of leptin, several studies have been conducted (Bogusiewicz et al., 2006; Carino et al., 2008; Zhou et al., 2015; Daley-Brown et al., 2019). These findings indicated that leptin, a known mitogenic, inflammatory, and angiogenic factor promoted the development of endometrial cancer mainly through the activation of classical biological signalling pathways.

**FIGURE 1:**
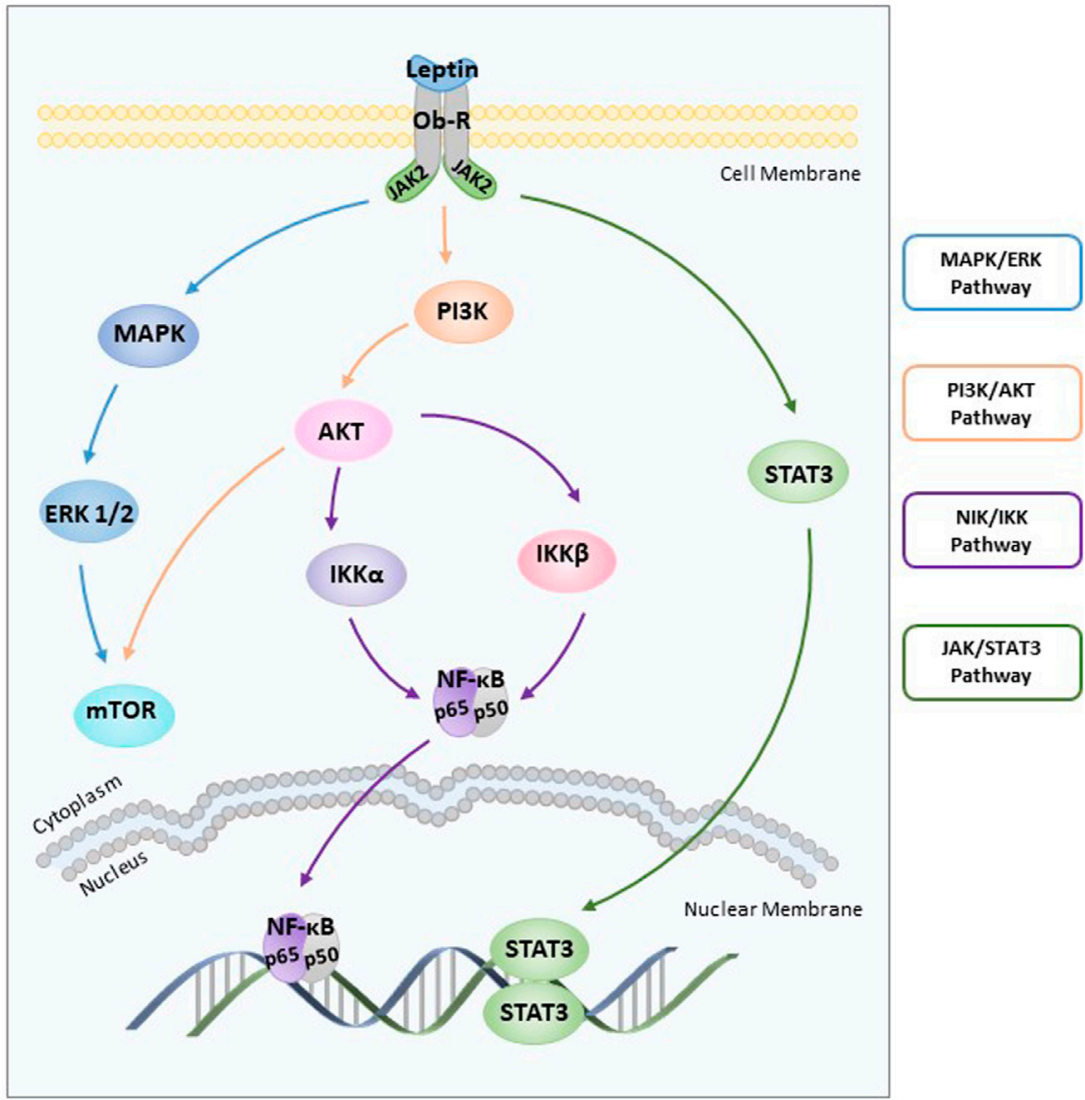
Role of leptin in endometrial cancer. Leptin induces two key cell-growth signalling pathways (ERK and AKT) after rapidly activating the JAK/STAT3 pathway. Leptin-induced NF-κB activation inhibits cancer cell apoptosis through the NIK/IKK signalling pathway. ERK: extracellular signal-regulated kinase; AKT: the serine/threonine kinase; JAK: janus-activated kinase; STAT3: signal transducers and activators of transcription 3; NIK: nuclear factor-kappa B inducing kinase; IKK: IKB kinase.

Leptin receptors, including both long and short receptors, can bind to janus-activated kinases and transduce certain signals (Hegyi et al., 2004). Leptin induces two key cell-growth signalling pathways (extracellular signal-regulated kinase (ERK) (Carino et al., 2008) and the serine/threonine kinase (AKT) (Carino et al., 2008)) after rapidly activating the janus-activated kinase (JAK)/signal transducers and activators of transcription (STAT) pathway (Hegyi et al., 2004) (Figure 1). The addition of tyrphostin AG490 abolished leptin-induced proliferation by blocking ERK and AKT phosphorylation (Sharma et al., 2006). Furthermore, the increased phosphorylation of ERK1/2 and leptin-induced stimulation of proliferation were observed upon treatment with 100 ng/ml leptin (Gong et al., 2007). Leptin triggers the phosphatidylinositol3-kinase (PI3K)/AKT pathway by activating the leptin receptor, which is correlated with cell proliferation and invasiveness (Bogusiewicz et al., 2006).

Another pathway involved in cancer progression is nuclear factor-kappaB inducing kinase (NIK)/IKB kinase (IKK), and leptin-induced NIK/IKK phosphorylation inhibits cancer cell apoptosis in carcinoma cells (Zhou et al., 2015) (Figure 1). First discovered in 1986, NF-κB is essentially involved in driving immune and inflammatory responses (Sen and Baltimore 1986). The NF-κB family includes five members and mediates DNA contact by forming homo or heterodimers (May and Ghosh 1997; Caamano and Hunter 2002). The IκB family of proteins, which includes four members, binds to the NF-κB family of proteins to inhibit the activity of transcription factors (Hoesel and Schmid 2013). Nuclear positivity for subunits of NF-κB as well as cytoplasmic staining for three IκB family members was assessed in 57 endometrial carcinoma cases by immunohistochemical evaluation. These data suggest that NF-κB/IκB may be involved in endometrial carcinoma cell proliferation and apoptosis (Pallares et al., 2004). Furthermore, leptin inhibits apoptosis of cancer cells by stimulating phosphorylation of IκBα, IκB kinase α (IKKα), IκB kinase β (IKKβ), and NIK in a dose-dependent manner (Zhou et al., 2015).

Leptin is involved in endometrial carcinoma cell mitosis, and leptin-mediated effects on endometrial cancer cell cycle progression are concentration-dependent. Leptin reduces the fraction of G0/G1-phase cells and increases S-phase cells by stimulating cyclin D1, a significant cell cycle regulator. Leptin-induced cyclin D1 overexpression increases STAT3-DNA and cAMP-response element binding protein (CREB)-DNA binding activity and recruitment (Catalano et al., 2009).

A positive correlation between overexpression of leptin and hypoxia-inducible factor 1 alpha (HIF-1α), an indicator of tissue hypoxia consisting of two subunits, was clearly observed in endometrial cancer tissues (Koda et al., 2007). Furthermore, leptin overexpression was stimulated through HIF-1α interaction with the leptin gene promotor in hypoxic adipocytes (Grosfeld et al., 2002). Among 48 human endometrioid adenocarcinoma patients, the number of patients positive for STAT3, HIF-1, leptin, and ObR was 36, 38, 29 and 15, respectively. It was clearly demonstrated that leptin induced HIF-1α through STAT3 in response to hypoxia (Wincewicz et al., 2008).

Leptin stimulates cell proliferation by increasing cyclooxygenase-2 (COX-2) protein expression through the JAK2/STAT3, MAPK/ERK, and PI3K/AKT signalling pathways (Gao et al., 2009). COX-2, a rate-limiting enzyme, is of considerable functional importance (Tsujii et al., 1998). The findings of basic *in vivo* and *in vitro* studies suggest that COX-2 overexpression is associated with increased susceptibility to endometrial cancer (Chen and Liao 2009; Ma et al., 2015). Increased COX-2 expression was found in higher-grade tumours. Several studies have indicated that functional activation of COX-2 is mediated by JAK2/STAT3 (Peng-Fei et al., 2021), MAPK/ERK (Adderley and Fitzgerald 1999), and PI3K/AKT (Rodriguez-Barbero et al., 2006) signalling pathways. After being treated with inhibitors (AG490, U0126, LY294002, and NS398) respectively, stimulated endometrial cancer cell proliferation and increased COX-2 protein expression induced by leptin were abolished (Gao et al., 2009). Therefore, COX-2 is also considered a significant biomarker for endometrial cancer diagnosis and prognosis (Oplawski et al., 2020).

Leptin-induced aromatase P450 (P450arom) overexpression increases oestrogen formation to promote endometrial cancer progression. P450arom, a key enzyme, is involved in the conversion of androstenedione to oestrogens (Noble et al., 1996). Excessive P450arom activity and transcript levels were found in endometrial cancer tissues. Higher P450arom mRNA and protein expression as well as oestradiol concentrations were observed in endometrial carcinoma cells treated with 100 ng/ml leptin, indicating a strong correlation between leptin and P450arom (Liu et al., 2013).

#### 3.1.2 Adiponectin

Adiponectin, a type of insulin-sensitizing adipokine, is secreted predominantly by WAT (Scherer et al., 1995; Hu et al., 1996; Maeda et al., 1996; Nakano et al., 1996). In addition, recent studies have indicated that adipose-derived stem cell (ASC) is an important source of intracellular adiponectin (Ciortea et al., 2018). In human plasma, Acrp30, a type of full-length adiponectin which consists of 247–amino acid protein is the main adiponectin form found in circulation (Scherer et al., 1995). In a large case‒control study, three SNPs in the ADIPOQ gene (rs3774262, rs1063539, rs12629945) were identified that potentially correlated with energy intake (Chen et al., 2012). Structurally, the adiponectin receptor has two isoforms, both of which include an internal N and an external C-terminus region (Yamauchi et al., 2003). AdipoR1 is ubiquitously expressed but has a higher affinity for globular adiponectin. However, AdipoR2 exhibits intermediate affinity for both globular and full-length adiponectin (Goldstein and Scalia 2004; Kadowaki and Yamauchi 2005). Analysis of endometrial tissues showed that both adiponectin receptors were expressed throughout the menstrual cycle and were especially present at higher levels in the mid-luteal phase (Takemura et al., 2006). Similar to leptin, adiponectin is also correlated with obesity. Higher levels of abdominal fat were found in the endometrial cancer group, and plasma adiponectin level was in a negative linear correlation with the abdominal fat level. (Mihu et al., 2013). Of note, significantly lower adiponectin levels were implicated in endometrial cancer patients (Rzepka-Gorska et al., 2008). Additionally, the abnormal expression of adiponectin receptors was observed in several insulin resistance-related tumours, such as breast cancer (Mocino-Rodriguez et al., 2017; Cicekdal et al., 2020), prostate cancer (Kaklamani et al., 2011; Huang et al., 2021), ovarian cancer (Jin et al., 2016), and endometrial cancer (Petridou et al., 2003; Soliman et al., 2006; Barb et al., 2007). In addition, adiponectin suppresses endometrial cancer proliferation by acting through AdipoRs, which were expressed in both tissue samples and cell lines (Moon et al., 2011). Positive staining was observed in low-grade adenocarcinoma, whereas negative staining was noted in high-grade adenocarcinoma. These results indicate that lower AdipoR expression was strongly correlated with higher histological grade in endometrioid adenocarcinoma (Yamauchi et al., 2012). Data from a study including 60 patients indicated that AdipoR1 levels are related to myometrial invasion (Yunusova et al., 2015). Moreover, another study indicated that the expression of AdipoR-1, not AdipoR-2, exerts suppressive effects on cancer cell proliferation, adhesion, and growth in a group of endometrial carcinoma patients (Yabushita et al., 2014).

Adiponectin directly reduced the viability of normal human endometrial stromal cells without any change in AdipoR1 and AdipoR2 levels (Bohlouli et al., 2013). Moreover, numerous findings showed that serum adiponectin levels were reduced in endometrial cancer patients compared with individuals with no history of endometrial cancer (Soliman et al., 2006; Cust et al., 2007; Ma et al., 2013; Zeng et al., 2015; Ellis et al., 2020). The expression levels of adiponectin and vaspin, which are considered anti-inflammatory molecules, are inversely proportional to endometrial cancer risk even after controlling for potential confounders (Erdogan et al., 2013). In particular, a linear dose-response relationship indicated that the risk was reduced by 3% for every 1 μg/ml increase in adiponectin (Lin et al., 2015). Furthermore, among women younger than 65 years, the odds ratios derived from three different models by multiple logistic regression indicated that the risk was reduced by 50% for a 1 SD increase in adiponectin (Petridou et al., 2003). Additionally, adiponectin concentrations were progressively reduced from grade 1 (19.04 μg/ml) to grade 2 (13.48 μg/ml), and finally grade 3 tumours (12.86 μg/ml). A significant difference was noted between grade 1 and grade 3 tumours but not between grade 1 and grade 2 tumours (Rzepka-Gorska et al., 2008).

Leptin-to-adiponectin ratios (L/A ratios) may be more informative in studies of the risk of endometrial cancer among postmenopausal women (Dallal et al., 2013). Higher L/A ratios were strongly related to endometrial cancer progression even after controlling for the factors of diabetes mellitus and age. The OR of the L/A ratio [6.0 (95% CI: 3.2–11.9)] was higher than those of leptin alone [3.2 (95% CI: 1.8–5.8)] or adiponectin alone [0.5 (95% CI: 0.3–0.9)], suggesting that L/A ratios in individuals may better indicate cancer growth and proliferation (Ashizawa et al., 2010).

**FIGURE 2:**
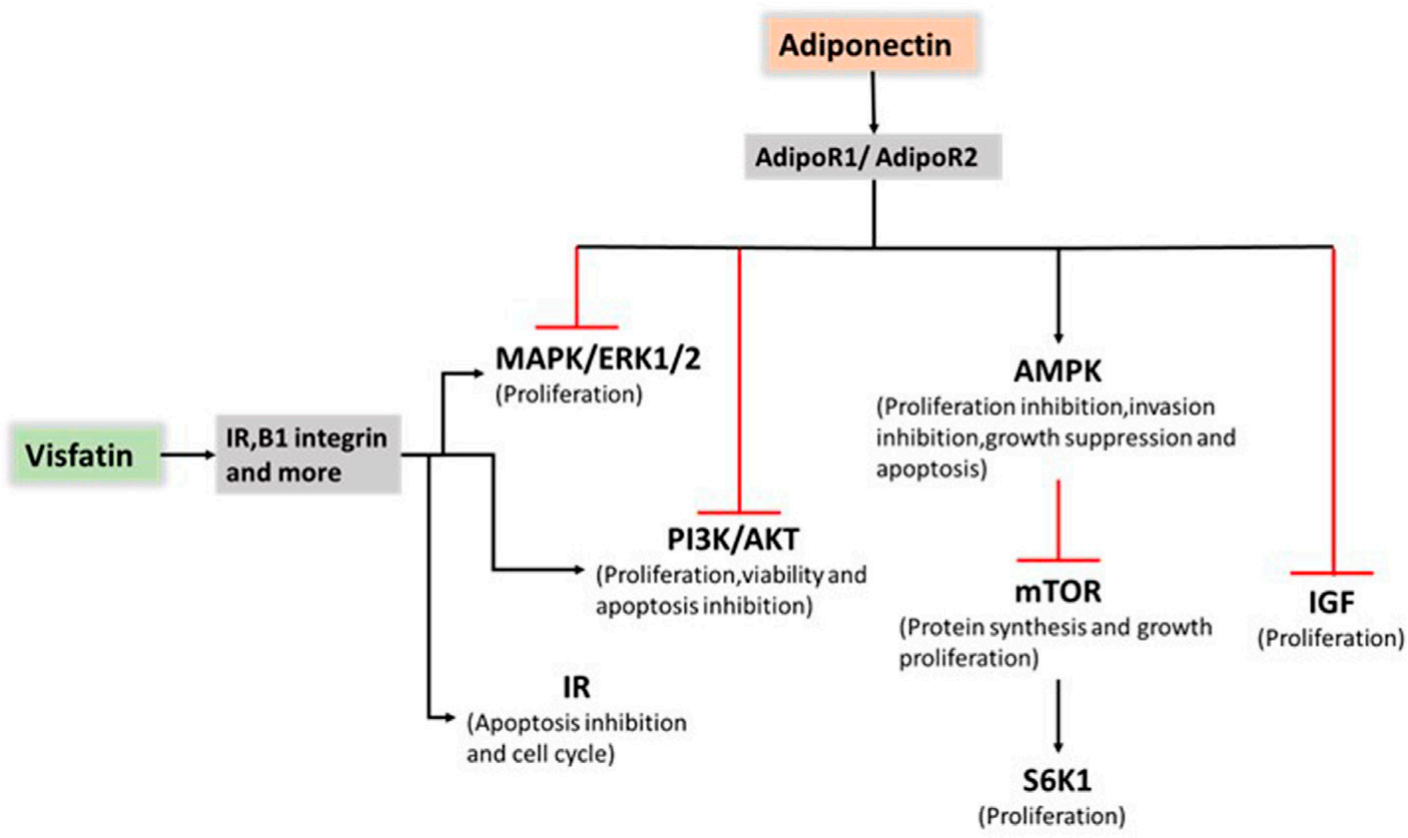
Role of adiponectin and visfatin in endometrial cancer progression. Adiponectin exerts an antiproliferative effect on endometrial cancer by stimulating AMPK pathway activation and suppressing PI3K/AKT, MAPK/ERK1/2 and IGF pathway activation. Visfatin promoted cancer progression mainly through PI3K/AKT, and MAPK/ERK1/2 pathway activation and IR. AMP: 5 adenosine monophosphate; AMPK: AMP-activated protein kinase; PI3K: phosphatidylinositol 3-kinase; AKT: the serine/threonine kinase; MAPK: mitogen-activated protein kinase; ERK1/2: extracellular signal-regulated kinases 1 and 2; IGF: insulin-like growth factor; IR: insulin resistance.

Adiponectin exerts an antiproliferative effect on endometrial cancer by increasing the number of G1/G0-phase cells and decreasing the number of S-phase cells. The reduction in cell counts in the HEC-1-A and RL95-2 cell lines reached approximately 30% and 20%, respectively, upon treatment with 40 mg/ml adiponectin. Furthermore, cyclin D1 and cyclin E2 expression was reduced, and 5 adenosine monophosphate-activated protein kinase (AMPK) was rapidly activated within 30 min in human endometrial cancer cell lines (Cong et al., 2007). Moon, H. S et al. (Moon et al., 2011) showed for the first time that adiponectin upregulated the tumour suppressor gene liver kinase B1 (LKB1), an adaptor molecule required for AMPK activation, to stimulate the AMPK/S6 axis. In addition, Wu et al. (Wu et al., 2012) demonstrated that Acrp30 effectively reduced leptin-induced STAT3 phosphorylation by stimulating the MAPK pathway in aggressive SPEC-2 endometrial cancer cells. After Ishikawa cells were treated with 10 μg/ml adiponectin, AMPK phosphorylation was rapidly activated and reached a maximum at 30 min. A 50% reduction in activated ERK and a 40% reduction in AKT expression were observed. Moreover, compound C inhibited adiponectin-induced ERK and AKT phosphorylation, demonstrating that ERK and AKT are downstream targets of AMPK. In addition, 10 μg/ml adiponectin treatment also caused significant reductions in cyclin D1 mRNA (49%), cyclin D1 protein (62%), B-cell lymphoma-2 (Bcl-2) mRNA (45%) and Bcl-2 protein (36%). This result suggested that adiponectin induced mitochondrial dysfunction by decreasing the Bcl-2/bcl-2-associated x (Bax) ratio (Zhang et al., 2015). Cai et al. (Cai et al., 2018) showed that AMPK phosphorylation was significantly induced by adiponectin, whereas mTOR and ribosomal protein S6 kinase-1 protein phosphorylation was inhibited. A considerably reduced proliferation inhibition ratio and enhanced cell migration were found in the inhibitor + adiponectin group than in the adiponectin group without the addition of an inhibitor. Adiponectin may inhibit cell proliferation and migration through the AMPK/(mTOR)/(S6K1) signalling pathway in patients with malignancies (Figure 2).

However, contrary to the previous role of adiponectin in suppressing cancer progression, several studies have showed that adiponectin contributes to an increased risk of liver cancer (Aleksandrova et al., 2014). Moreover, a study involving in exploring the relation between cancer and adiponectin underlying the obesity paradox, has showed that exogenous adiponectin significantly inhibited cell apoptosis by up-regulating p-AMPK and Bcl-xL levels in renal cell carcinoma (Ito et al., 2017). This conclusion was consistent with the results of the later study conducted by Lee et al. (Lee et al., 2020). The study conducted in Hong Kong, including 5658 participants, indicated an interesting adiponectin paradox. They demonstrated that higher adiponectin concentrations might be harmful, and positively correlated with the incidence and deaths of cancer in type 2 diabetes (Lee et al., 2020). As the role of adiponectin still remains controversial in various cancers, further studies should be directed to exploring the complex mechanism.

#### 3.1.3 Visfatin

Visfatin, a 52 kDa protein (Fukuhara et al., 2005), plays a significant role in cell growth (Zhang et al., 2011) and insulin resistance (Fukuhara et al., 2005). Recently, accumulating evidence has suggested that visfatin may be a complementary diagnostic and prognostic marker for malignancies, especially those that are strongly related to dysfunctional adipose tissue, such as breast cancer (Rajput et al., 2022), colorectal cancer (Zhao et al., 2020), and endometrial cancer (Mu et al., 2012). Tian et al. (Tian et al., 2020) reported that visfatin protein expression was upregulated by the PI3K/AKT and MAPK/ERK signalling pathways in polycystic ovary syndrome (PCOS) patients with endometrial cancer.

Tian et al. (Tian et al., 2013) suggested that serum visfatin levels were significantly higher in endometrial cancer patients compared with other groups. Furthermore, visfatin expression was measured in tissue samples. Visfatin tissue expression increased gradually from normal proliferative or secretory endometrium (58.1%) and hyperplastic endometrium (66.7%) to endometrial cancer (80.5%). Moreover, visfatin expression was significantly related to serum levels in 50 endometrial cancer patients. High serum visfatin levels represent a key factor correlated with deep myometrial invasion and poor survival (Tian et al., 2013). Visfatin promotes cancer progression mainly through PI3K/AKT and MAPK/ERK1/2 activation as well as insulin resistance (IR) (Figure 2). In 2014, Nergiz Avcioglu et al. (Nergiz Avcioglu et al., 2015) indicated three possible mechanisms (obesity, increased lipolysis, and insulin resistance) to explain the increased serum visfatin levels in endometrial cancer (Nergiz Avcioglu et al., 2015). A study focusing on the molecular mechanisms showed that visfatin exerts pro-proliferative and anti-apoptotic effects by stimulating cell proliferation and increasing the S-phase fraction of cells.

The expression of visfatin and its substrates was upregulated in the context of IR, and maximal levels were noted at 30 min. Increased C-MYC and cyclin D1 expressions as well as decreased caspase-3 expression were also observed with visfatin treatment. To confirm the effect of the PI3K/AKT and MAPK/ERK signalling pathways, Ishikawa cells were treated with 400 ng/ml visfatin. Larger G1 and S-phase fractions were found in Ishikawa cells pretreated with the inhibitor (Wang et al., 2016). Similar results are presented by Cymbaluk-Ploska et al. (Cymbaluk-Ploska et al., 2018). The visfatin concentration was 15.9 ng/ml for the endometrial cancer group and 9.5 ng/ml for the other. Furthermore, a slightly higher visfatin concentration was noted for cases with lower histological differentiation (22.2 and 31.8 ng/ml) compared with well-differentiated cases (17.3 and 22.2 ng/ml). The visfatin level was inversely proportional to the overall survival (OS) of patients (Cymbaluk-Ploska et al., 2018). A retrospective case‒control study showed that the visfatin-adiponectin ratio in 53 endometrial cancer patients was significantly higher than that in the control group (Wang et al., 2019).

#### 3.1.4 Galectin

It is clear that galectins are integrated into the physiological and pathological systems of individuals with a wide range of biological functions (Liu et al., 2002). To date, 11 identified different subtypes have been classified into three subgroups according to structure (prototype, tandem repeat-type, and chimeric-type) (Chou et al., 2018). Among them, four forms (galectin-1, galectin-3, galectin-7, and galectin-9) have been closely linked to gynecological cancer cell biology and immunology (Chetry et al., 2018). Furthermore, multiple studies have indicated that galectin-1, a homodimeric protein involved in angiogenesis (Thijssen and Griffioen 2014) and cross-linking receptors (Hernandez et al., 2006), and galectin-3, a chimaera-type protein associated with cancer metastasis (Fortuna-Costa et al., 2014) and inflammatory regulation (Henderson and Sethi 2009), are mainly involved in endometrial cancer. Galectin-1 expression was observed in endometrioid endometrial adenocarcinoma (EA) tissue (Zinovkin et al., 2019). Higher galectin-1 expression suggested a poorer prognosis (Sun and Dai 2020). In addition, galectin-1 immunoreaction was positively proportional to endometrial cancer grade, increasing from G1 to G3 (Mylonas et al., 2007). The microcystic, elongated and fragmented (MELF) pattern was inversely proportional to endometrial cancer patient survival (Stewart et al., 2009; Zinovkin et al., 2017). The median level of galectin-1 expression among 49 subjects was obviously higher (78.6%) in the positive group. The statistically significant differences analyzed by the Mann‒Whitney test additionally indicated that this marker may be of considerable functional importance in the OS of patients (Zinovkin et al., 2019).

Galectin-1 and galectin-3 immunoperoxidase staining of the uterine carcinoma specimens obtained from Duke University Medical Center was performed and statistically analyzed. Lower scores of galectin-1 expression were found in normal endometrium (scores from 0 to 2), whereas higher scores were found in endometrial carcinomas (scores from 1 to 3). In contrast, galectin-3 expression was significantly decreased in endometrial cancer (van den Brule et al., 1996). This conclusion was consistent with the results of a later study conducted in the Middle East. This finding demonstrated that galectin-3 may play a role in the suppression of cancer progression. Galectin-3 immunoreactivity progressively decreased from normal samples (80%) to endometrial carcinoma (55%), indicating poor prognoses (Al-Maghrabi et al., 2017). Interestingly, deeper invasion of the myometrium was found in cancer cells with only cytoplasmic immunoreactivity (van den Brule et al., 1996). The extent, intensity, and immunohistochemical reactivity of epithelial and stromal galectin-3 expression were reduced in the cancer group. The percentage of the cases with lymph node metastasis negative for galectin-3 expression (64%) was increased almost four-fold compared with cases without lymph node metastasis (18%). This investigation suggests that galectin-3 may be involved in the pathogenesis of endometrial carcinomas and lymph node metastasis (Ege et al., 2011).

However, contradictory results from a study involving 144 patients showed that increased galectin 3 expression was observed in patients with lymphovascular space invasion (Cymbaluk-Ploska., et al., 2020). The mean scores progressively increased from normal endometria (2.58) and atypical hyperplasia (4.77) to clear cell carcinoma (6.71), and significant differences were noted among the various conditions. Based on these findings, Brustmann et al. (Brustmann et al., 2003), assumed that galectin-3 expression was essential to maintain a transformed phenotype in endometrial carcinoma (Brustmann et al., 2003). To investigate the effect of galectin-3 on the endometrial cell cycle and adhesion, multiple analysis methods were used. After seventy-two hours of galectin-3 siRNA transfection, galectin-3 mRNA and protein expression were reduced by 70%–90% in RL95-2 cells. A decrease in S-phase cells and an increase in G1-phase cells were observed. Thus, galectin-3 may be involved in promoting cell adhesion and increasing integrin expression (Lei et al., 2007). Additionally, considering the fact that the environment composed of numerous adipokines and cytokines that promote tumour growth, the different conclusions may be clarified by method sensitivity, case differences, treatment differences, and different sizes of samples. To better understand the effect of galectin-3 and related biological signalling pathways on tumour size, growth, characteristics, and malignancy in endometrial cancers, more studies, such as longitudinal studies and large-scale studies, are needed.

### 3.2 Role of adipose-secreted inflammatory cytokines in endometrial cancer progression

Inflammatory cytokines, such as interleukin-1β (IL-1β), interleukin-6 (IL-6), and interleukin-8 (IL-8), can modify the immunological network in the endometrium. The primary sources of inflammatory cytokines mainly include inflammatory cells, adipocytes, and cancer cells. Among them, IL-1β and IL-6 are secreted by adipocytes through endocrine and paracrine secretion and are correlated with a modified adipocyte phenotype (Dirat et al., 2011). Inflammatory cytokines are the key factors that explain the difference in the immune microenvironment between normal and malignant endometria. Therefore, understanding the role of inflammatory cytokines in proinflammatory and protumorigenic effects on endometrial cancer progression is crucial.

IL-1β, IL-6, and IL-8, which exhibit a wide range of complex functions, have been extensively examined. Notably, IL-1 is ubiquitously expressed in endometrial tissues (Van Le et al., 1991). However, data from a clinical study revealed a significant increase in IL-8 concentrations, not IL-Iβ and IL-6, which were too low to detect (Chopra et al., 1997). Furthermore, later experiments clearly demonstrated that leptin significantly increased the levels of IL-1 and interleukin-1 receptor tI (IL-1R tI) in a dose-dependent manner. Based on experiments using a kinase inhibitor, the results indicated that leptin-mediated activation of the JAK2/STAT3, PI3K/AKT1, and mTOR signalling pathways was associated with an increase in IL-1β levels in primary endometrial epithelial cells. In contrast, leptin induced IL-1R tI in all endometrial epithelial cells through leptin canonical signalling pathways that generally include JAK2/STAT3, MAPK/ERK1/2, and mTOR without PI3K/AKT1 involvement (Carino et al., 2008).

Adiponectin also stimulated AMPK phosphorylation and suppressed the secretion of IL-6 and IL-8 induced by IL-1β in human endometrial stromal cells (ESCs), suggesting the effect of adiponectin on regulating energy supply and anti-inflammatory function (Takemura et al., 2006).

**FIGURE 3:**
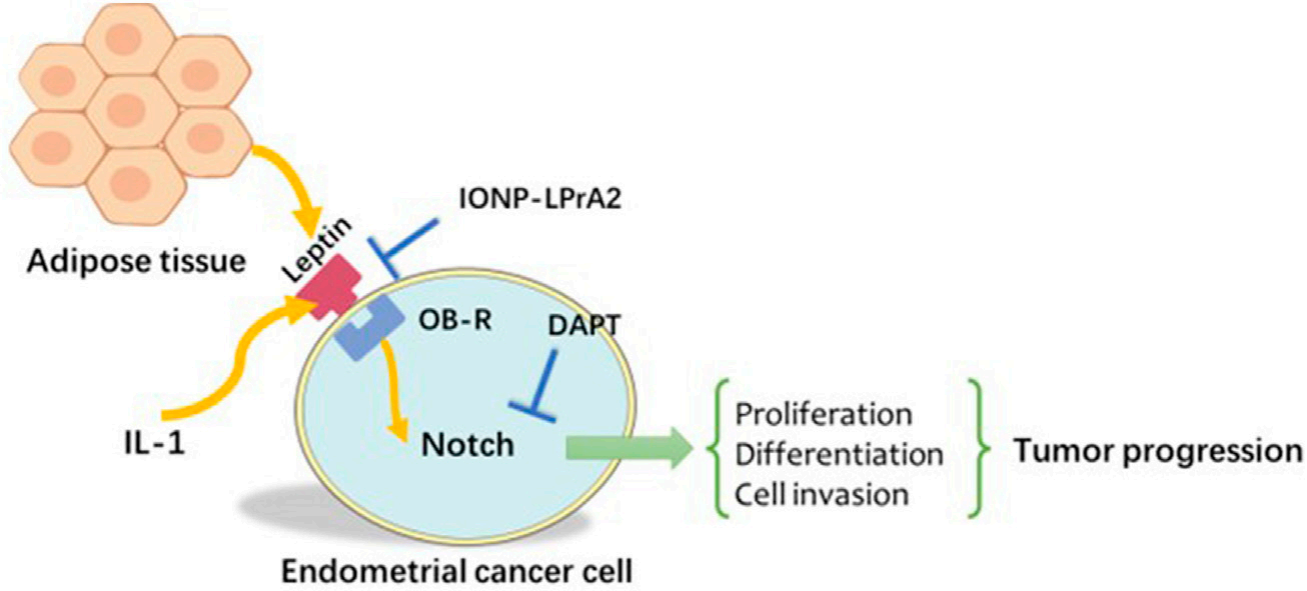
Notch, IL-1, and leptin crosstalk outcome (NILCO). Leptin induced Notch receptor, ligand, and targeted molecule expression. The inhibition of Notch and IL-1 signalling *in vitro* reduced leptin-induced invasion. IL-1: Interleukin-1.

When assessing endometrial cancer cells using cell invasion assays and statistical analysis, Lipsey et al. (Lipsey et al., 2016) found that Notch, IL-1, and leptin crosstalk outcome (NILCO) was more highly expressed in type II endometrial cancer, the more aggressive form, not type I. Moreover, leptin-induced invasion of endometrial carcinoma cells was significantly reduced in the presence of an inhibitor (Daley-Brown et al., 2017). Remarkably, the levels of Notch receptors, ligands, and targeted molecules were at least a twofold increase compared to basal culture conditions without leptin treatment. After DAPT and anti-IL-1R tI antibodies were added, the results showed that leptin-induced migration of malignancies was abrogated. The role of leptin was more prominent in the more malignant phenotype, such as the more aggressive and poorly differentiated An3CA endometrial cancer cell line. Leptin-induced NILCO molecules in endometrial cancer affect cell proliferation, aggressiveness, and chemoresistance (Daley-Brown et al., 2019). Taken together, these studies indicated the complex crosstalk among Notch, IL-1, and leptin as well as the involvement of IL-1 in inducing inflammatory progression and upregulating leptin expression in endometrial cancer (Figure 3).

### 3.3 Role of adipose-secreted angiogenic factors in endometrial cancer progression

Adipose-secreted angiogenic factors, such as vascular endothelial growth factor (VEGF) and fibroblast growth Factor 21 (FGF21), play significant roles in stimulating angiogenesis and forming the proangiogenic microenvironment. Potential therapeutic implications targeting VEGF and FGF21 may open up new avenues for endometrial cancer women with cancer cell metastasis.

VEGF, a multiple proangiogenic factor observed across endometrioid endometrial adenocarcinoma (EA) cells in different stages, was correlated with abnormal vasculature formation, insulin sensitivity, and adipocyte death (Sun et al., 2012; Goel and Mercurio 2013). Moderate VEGF expression was positively correlated with EA progression as well as an elongated and fragmented (MELF) pattern. However, this parameter was inversely proportional to the number of survival days (Zinovkin et al., 2019). A preliminary study suggested that leptin significantly increased the levels of VEGF and vascular endothelial growth factor receptor 2 (VEGFR2) through the MAPK/ERK1/2 and mTOR signalling pathways (Carino et al., 2008). In addition, overexpression of VEGF and its receptors in uterine tissue appeared to be affected by cotreatment (leptin and oestradiol) probably through the ERK1/2 and STAT3 pathways (Shetty et al., 2020). Interestingly, additional experiments clearly showed that leptin-induced angiogenesis was probably attributed to activating VEGFR-Notch signalling crosstalk in overweight cancer patients with increased expression of VEGF, VEGFR-2, and Notch (Lanier et al., 2016).

Fibroblast growth Factor 21 (FGF21) belongs to the sixth subfamily of FGFs and mainly modulates the storage of carbohydrates (Beenken and Mohammadi 2009). Based on comparative analysis, high FGF21 concentrations were positively related to high leptin levels. Taken together, the results showed that FGF21 concentrations were higher in poorly and moderately differentiated tumours compared with highly differentiated tumours. In addition, the area under the receiver operator characteristic curve (AUC) for FGF21 was 0.81, indicating that FGF21 was a promising diagnostic biomarker with good sensitivity and specificity through FGFR 2 and the PI3K/AKT and mTOR signalling pathways (Cymbaluk-Ploska et al., 2020).

### 3.4 Role of other adipocytokines in endometrial cancer progression

On review of the recent studies, other identified adipocytokines, including plasminogen activator inhibitor-1 (PAI-1) (Wang et al., 2021), and fatty acid-binding protein 4 (FABP4) (Wu et al., 2021), also play important roles in regulating various physiological processes. Of note, these adipocytokines have considerable consequences for promoting the proliferation and migration of endometrial cancer cells and may be possible targets for the therapy.

PAI-1, a promising prognostic factor involving in selective degradation of extracellular matrix components (Andreasen et al., 1997; Fredstorp-Lidebring et al., 2001), has been found to be associated with neovascularization, invasion, and migration in breast (Schmitt et al., 2010), prostate (Almasi et al., 2011), colorectal (Markl et al., 2017), ovarian (Zhang et al., 2013), and endometrial cancers (Tecimer et al., 2001). Women with PAI-1 rs1799889 4G/4G genotype are more likely to be at risk for endometrial cancer and the susceptibility to cancer may be associated with the 4G allele. (Su et al., 2011; Xu et al., 2012). Compared to normal endometrium, concentrations of PAI-1 in cytosols of endometrial cancer were significantly higher (Kohler et al., 1997; Osmak et al., 2001). In addition, expression of PAI-1 was regulated by estrogen and progesterone, and appeared negatively correlated with estrogen and progesterone receptor levels (Fujimoto et al., 1996; Steiner et al., 2008). The potential of sex steroids-dependent metastasis plays significant roles in cancer progression (Gotte et al., 2010; Fujimoto and Sato 2011). Previous studies showed that PAI-1 was positively correlated with cancer stage, but negatively correlated with relapse free time and OS of patients (Tecimer et al., 2001; Steiner et al., 2008). As one of the most abundant adipocytokines in adipose stromal cells (ASCs), PAI-1 could diminish transforming growth factor β (TGF-β)-mediated tumor suppressor activity through the TGF-β/SMAD pathway (Lin et al., 2020).

FABP4, belonging to the fatty acid binding proteins (FABPs) family, has a central role in tumour metastasis and endothelial migration by regulating metabolic and inflammatory pathways (Hotamisligil and Bernlohr 2015). As a marker involved in adipocyte differentiation (Bernlohr et al., 1984), FABP4 promotes the progression of feminine cancers, such as ovarian cancer, and cervical cancer (Gharpure et al., 2018; Jin et al., 2018). However, a recent study showed that FABP4 might play a possible suppressive role in endometrial cancer cell proliferation, migration, and invasion through the PI3K/AKT pathway (Wu et al., 2021). These studies have showed that the effects FABP4 exerts on cancers may be related to tumor type and signaling pathways. To further explore the decreased expression of FABP4 in endometrial cancer, more researches are required.

### 3.5 Possible role of adipocytokines in the treatment of endometrial cancer

**FIGURE 4:**
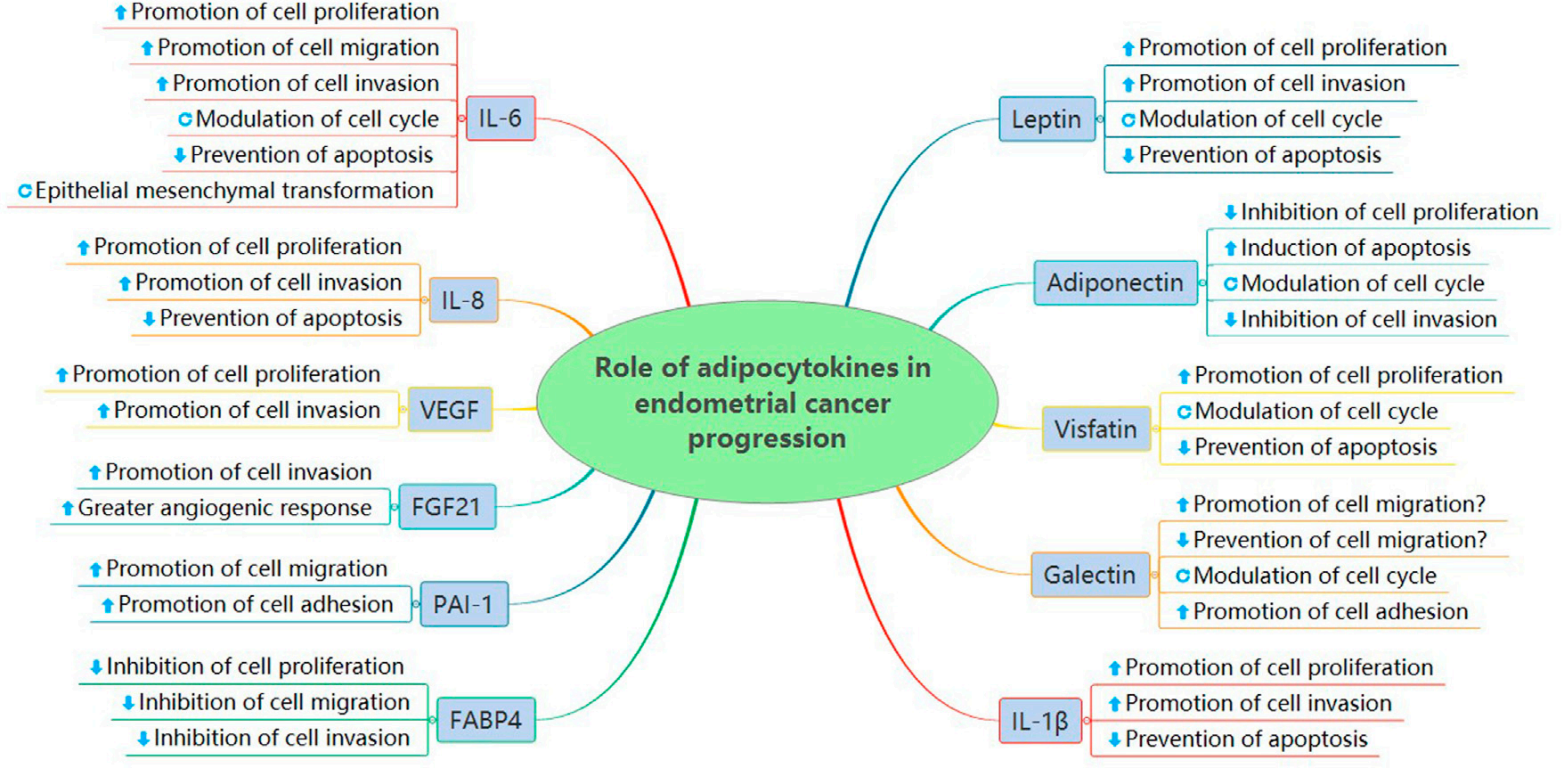
Role of adipocytokines in endometrial cancer progression.

Adipocytokines, including adipokines, inflammatory cytokines, and angiogenic factors, are significant biomarkers in various cancers, particularly endometrial cancer. To date, among the identified adipocytokines, some have been found to be good prognostic factors with a wide range of biological functions, including suppression of cell proliferation, induction of apoptosis, and reduced cell invasion. However, other adipocytokines, such as leptin, galectin, and visfatin, are considered poor prognostic factors associated with the promotion of cell proliferation, inhibition of apoptosis, and increased cell invasion (Figure 4). Leptin and adiponectin are the two main adipocytokines involved in most studies in endometrial cancer. However, studies on the potential molecular mechanisms of other adipocytokines, such as resistin, galectin, and visfatin, are limited. Particularly, contradictory results have been reported from different studies on galectin-3 concentrations and expression in endometrial cancers. The main inflammatory pathways predominantly reported include the MAPK/ERK1/2, JAK/STAT3, PI3K/AKT/mTOR, Notch, IR, IGF, AMPK/ERK, and AKT signalling pathways. When using specific inhibitors, endometrial cancer cell proliferation, invasion, and migration were reduced.

Based on the roles of significant adipocytokines and specific inhibitors, the use of targeted treatments in cancers has been studied in various experiments. Metformin, a potential anti-cancer drug, could induce cell cycle arrest and apoptosis through the AMPK and mTOR signalling pathways (Jalving et al., 2010). Furthermore, compared to metformin (Met) alone, metformin and silibinin (Sil) in magnetic PLGA/PEG nanoparticles (NPs) kill lung cancer cells more rapidly by reducing the expression of leptin and its receptor (Salmani Javan et al., 2022). Thiazolidinediones (TZDs) (rosiglitazone, pioglitazone) are also reported to increase adiponectin levels and decrease leptin, tumor necrosis factor-α (TNF-α), and IL-6 levels through modulatory mechanisms (Garikapati et al., 2019; Biondo et al., 2020). Moreover, TZDs have played important roles in preventing progression of hepatocellular carcinoma (HCC) (Arvind et al., 2021), colon cancer (Yoon et al., 2020), and lung cancer (Konieczna et al., 2015). Atorvastatin reduces cardiovascular mortality by increasing levels of adiponectin, which is involved in insulin resistance (Koh et al., 2005; Ando et al., 2008). Atorvastatin has been reported to be used as a kind of important therapy in oesophageal adenocarcinoma by suppressing leptin-induced activation of cdc42 and AKT (Beales and Ogunwobi 2021).

Furthermore, recent studies have showed that mild obesity (BMI≥ 25.0,≤ 29.9) is correlated to an improved immunotherapy response (Li and Kalantar-Zadeh 2013; Li et al., 2017). The cancers who have mild obesity are more likely to reach a balance between pro- and anti-inflammatory cytokines (Assumpcao et al., 2022). Compared with poor response to chemotherapy in obese patients (Horowitz and Wright 2015), immunotherapy may be a more favorable therapeutic approach for the obesity (Waldman et al., 2020). The dysregulation of the secretion of adipocytokines, which involves in T cell modulation, macrophage polarization, and binding of adipocyte PD-L1 to anti-PD-L1 antibodies, affects immune checkpoint inhibitor therapy (Assumpcao et al., 2022). By using checkpoint inhibitor (anti–CTLA-4 mAb), Murphy et al. (Murphy et al., 2018) have found that leptin was a contributor to the failure of tumor immunotherapy. It implicated the potential role of leptin in the efficacy of immunotherapy.

## 4 Conclusion

Adipocytokines, regulating various physiological and pathological processes, play crucial roles in endometrial cancer progression. Larger prospective studies focusing on adipocytokines and specific inhibitors, particularly immune checkpoint inhibitor therapy, should be directed at preventing the rapidly increasing prevalence of gynecological malignancies.
